# Supplementary material for: Resolving the full spectrum of human genome variation using Linked-Reads
Source: Genome Res. 2019 Apr;29(4):635–45. doi: 10.1101/gr.234443.118 (PMC6442396; doi:10.1101/gr.234443.118)
Supplement: Supplemental Material [file supp_gr.234443.118_Supplemental_longranger-2.2.zip › longranger-2.2.tar.bz2!/lr2.2-public/lib/stan/cmdstan/stan/doc/api/html/base__leapfrog_8hpp_source.html]

Stan: src/stan/mcmc/hmc/integrators/base\_leapfrog.hpp Source File


|  |  |
| --- | --- |
|  | Stan  2.5.0  probability, sampling & optimization |


- Main Page
- Related Pages
- Namespaces
- Classes
- Files

- File List
- File Members

All Classes Namespaces Files Functions Variables Typedefs Enumerations Enumerator Friends Macros Pages

- src
- stan
- mcmc
- hmc
- integrators

base\_leapfrog.hpp

Go to the documentation of this file.

1 #ifndef STAN\_\_MCMC\_\_BASE\_\_LEAPFROG\_\_BETA

2 #define STAN\_\_MCMC\_\_BASE\_\_LEAPFROG\_\_BETA

3

4 #include <iostream>

5 #include <iomanip>

6 #include <stan/mcmc/hmc/integrators/base\_integrator.hpp>

7

8 namespace stan {

9

10  namespace mcmc {

11

12  template <typename H, typename P>

13  class base\_leapfrog: public base\_integrator<H, P> {

14

15  public:

16

17  base\_leapfrog(std::ostream\* o): base\_integrator<H, P>(o) {};

18

19  void evolve(P& z, H& hamiltonian, const double epsilon) {

20

21  begin\_update\_p(z, hamiltonian, 0.5 \* epsilon);

22

23  update\_q(z, hamiltonian, epsilon);

24  hamiltonian.update(z);

25

26  end\_update\_p(z, hamiltonian, 0.5 \* epsilon);

27

28  }

29

30  void verbose\_evolve(P& z, H& hamiltonian, const double epsilon) {

31

32  this->out\_stream\_->precision(6);

33  int width = 14;

34  int nColumn = 4;

35

36  if (this->out\_stream\_) {

37

38  \*(this->out\_stream\_) << "Verbose Hamiltonian Evolution, Step Size = " << epsilon << ":" << std::endl;

39  \*(this->out\_stream\_) << " " << std::setw(nColumn \* width) << std::setfill('-')

40  << "" << std::setfill(' ') << std::endl;

41  \*(this->out\_stream\_) << " "

42  << std::setw(width) << std::left << "Poisson"

43  << std::setw(width) << std::left << "Initial"

44  << std::setw(width) << std::left << "Current"

45  << std::setw(width) << std::left << "DeltaH"

46  << std::endl;

47  \*(this->out\_stream\_) << " "

48  << std::setw(width) << std::left << "Operator"

49  << std::setw(width) << std::left << "Hamiltonian"

50  << std::setw(width) << std::left << "Hamiltonian"

51  << std::setw(width) << std::left << "/ Stepsize^{2}"

52  << std::endl;

53  \*(this->out\_stream\_) << " " << std::setw(nColumn \* width) << std::setfill('-')

54  << "" << std::setfill(' ') << std::endl;

55

56  }

57

58  double H0 = hamiltonian.H(z);

59

60  begin\_update\_p(z, hamiltonian, 0.5 \* epsilon);

61

62  double H1 = hamiltonian.H(z);

63

64  if (this->out\_stream\_) {

65

66  \*(this->out\_stream\_) << " "

67  << std::setw(width) << std::left << "hat{V}/2"

68  << std::setw(width) << std::left << H0

69  << std::setw(width) << std::left << H1

70  << std::setw(width) << std::left << (H1 - H0) / (epsilon \* epsilon)

71  << std::endl;

72

73  }

74

75  update\_q(z, hamiltonian, epsilon);

76  hamiltonian.update(z);

77

78  double H2 = hamiltonian.H(z);

79

80  if (this->out\_stream\_) {

81

82  \*(this->out\_stream\_) << " "

83  << std::setw(width) << std::left << "hat{T}"

84  << std::setw(width) << std::left << H0

85  << std::setw(width) << std::left << H2

86  << std::setw(width) << std::left << (H2 - H0) / (epsilon \* epsilon)

87  << std::endl;

88

89  }

90

91  end\_update\_p(z, hamiltonian, 0.5 \* epsilon);

92

93  double H3 = hamiltonian.H(z);

94

95  if (this->out\_stream\_) {

96

97  \*(this->out\_stream\_) << " "

98  << std::setw(width) << std::left << "hat{V}/2"

99  << std::setw(width) << std::left << H0

100  << std::setw(width) << std::left << H3

101  << std::setw(width) << std::left << (H3 - H0) / (epsilon \* epsilon)

102  << std::endl;

103

104  \*(this->out\_stream\_) << " " << std::setw(nColumn \* width) << std::setfill('-')

105  << "" << std::setfill(' ') << std::endl;

106

107  }

108

109  }

110

111  virtual void begin\_update\_p(P& z, H& hamiltonian, double epsilon) = 0;

112  virtual void update\_q(P& z, H& hamiltonian, double epsilon) = 0;

113  virtual void end\_update\_p(P& z, H& hamiltonian, double epsilon) = 0;

114

115  };

116

117  } // mcmc

118

119 } // stan

120

121

122 #endif

base\_integrator.hpp

stan::mcmc::base\_leapfrog::evolve

void evolve(P &z, H &hamiltonian, const double epsilon)

**Definition:** base\_leapfrog.hpp:19

stan::mcmc::base\_integrator::out\_stream\_

std::ostream \* out\_stream\_

**Definition:** base\_integrator.hpp:21

stan::mcmc::base\_leapfrog::base\_leapfrog

base\_leapfrog(std::ostream \*o)

**Definition:** base\_leapfrog.hpp:17

stan::mcmc::base\_leapfrog::update\_q

virtual void update\_q(P &z, H &hamiltonian, double epsilon)=0

stan::mcmc::base\_integrator

**Definition:** base\_integrator.hpp:11

stan::mcmc::base\_leapfrog::verbose\_evolve

void verbose\_evolve(P &z, H &hamiltonian, const double epsilon)

**Definition:** base\_leapfrog.hpp:30

stan::mcmc::base\_leapfrog

**Definition:** base\_leapfrog.hpp:13

stan::mcmc::base\_leapfrog::begin\_update\_p

virtual void begin\_update\_p(P &z, H &hamiltonian, double epsilon)=0

stan::mcmc::base\_leapfrog::end\_update\_p

virtual void end\_update\_p(P &z, H &hamiltonian, double epsilon)=0


---

[ Stan Home Page ]

*© 2011–2014,
Stan Development Team.*
